# Comparison of Cardiovascular Risk Estimation and Statin Prescribing in Primary Care: A Retrospective Cohort Study

**DOI:** 10.7759/cureus.95956

**Published:** 2025-11-02

**Authors:** Adam Merlo, Tania Rubaiyyat

**Affiliations:** 1 Family Medicine, Western University, London, CAN; 2 Family and Community Medicine, University of Toronto, Toronto, CAN

**Keywords:** acc/aha risk estimator, cardiac risk factors and prevention, cardiovascular prevention, guidelines in medicine, statin use

## Abstract

Introduction: Statin prescribing for primary prevention remains a topic of debate, especially among individuals with low to moderate risk for cardiovascular disease (CVD), partly due to limitations of current cardiovascular risk assessment tools. This study aimed to determine whether differences exist in risk estimation among various cardiovascular risk calculators used in Canadian clinical practice guidelines and to describe the proportion of patients who may fall into a different risk category if an alternative risk calculator were used.

Methods: This work was approved by the local research ethics board. A retrospective chart review was conducted for adult patients aged 40 and older without a statin-indicated condition or prior cardiovascular event who underwent lipid assessment at a single family medicine center in London, Ontario, between 2010 and 2023. Three online calculators and two risk estimators (Framingham and American Society for Cardiovascular Disease) were used to re-estimate cardiovascular risk and compare the results with the values documented in the patient’s chart.

Results: Of 50 patients, 20% did not have a documented cardiovascular risk value in their chart at the time of lipid assessment. However, the mean difference in Framingham risk values between the electronic medical record and the PEER (patients, experience, evidence, research) lipid online calculator was statistically significant (3.44%, 95% CI 0.11-6.76, p<0.05). Additionally, 23 (46%) patients would have fallen into a lower risk category according to Canadian clinical practice guidelines if the atherosclerotic cardiovascular disease (ASCVD) risk calculator had been used instead of the Framingham estimator for CVD risk assessment.

Conclusion: Cardiovascular risk percentages differ between those calculated using an electronic medical record tool and those calculated with online calculators. Depending on the tool used, a proportion of patients may fall into a different cardiovascular risk category, resulting in different management decisions. Specifically, patients who would fall into a lower risk category could be considered for lifestyle management alone rather than statin initiation. Further research is needed to guide consistent use of available point-of-care risk tools by clinicians, and clinical practice guidelines should incorporate these recommendations.

## Introduction

In Canada, screening for dyslipidemia remains one of the earliest forms of primary preventive practices in a patient’s lifespan, in part due to its strong correlation with the development of atherosclerosis, cardiovascular events, hospitalization, and death [1]. In 1948, the initial stages of the Framingham Heart Study were launched with over 5,000 participants, making it the first study of its size and caliber [2]. Major findings were first published in 1957, nearly one decade later [3]. The study was renowned for shifting the paradigm from treating individuals with established CVD to preventing disease in those at risk [1,4]. Later, the term “risk factors,” including hypertension, hyperlipidemia, and diabetes mellitus, was coined based on the Framingham study’s findings, which were then used to develop a risk calculator to measure an individual’s quantitative risk of developing a major cardiovascular event within a 10-year period [5-7].

The Canadian Cardiovascular Congress has used this evidence, given its large sample size and longevity, to inform clinical decision-making guidelines widely implemented by healthcare providers across the country [8,9]. The rationale for using this evidence is based on estimating absolute risk derived from large datasets representing population samples [8]. The risk estimation relies on group averages that are applied to individual patients in practice [8,9]. Although the Framingham risk score has been widely used for decades and has proven useful in predicting CVD risk in Western populations, it has been criticized for overestimating risk in others [10-12]. This is because the score was developed using data from a predominantly white, male cohort in Framingham, Massachusetts, and may not accurately reflect risk in other populations with differing demographic and lifestyle factors [10-21].

The Framingham risk score has been found to overestimate risk in women and ethnic minorities, who may have different risk factor patterns and CVD prevalence [14-23]. Additionally, the score does not account for important factors such as inflammatory or insulin resistance markers, which can affect CVD risk [10-21]. This may lead to overdiagnosis and overtreatment, as well as missed opportunities for prevention among individuals at lower risk than indicated. Consequently, as multiple studies have suggested overestimation of cardiovascular risk, the question arises as to whether this has led to overprescription of lipid-lowering agents in primary care settings.

Whether differences in risk estimation lead to the overprescription of statin medications remains debated. Some studies suggest that statins are overprescribed, particularly among individuals with low to moderate CVD risk, while others argue that the benefits of statins in reducing CVD risk outweigh the potential harms of overuse [10-23]. Factors contributing to this debate include cost-effectiveness, potential side effects, and limitations of current CVD risk assessment tools [9-11].

In the United States, the American Heart Association atherosclerotic cardiovascular disease (ASCVD) risk calculator is commonly used [24]. This tool was developed to account for individual differences, including those among ethnic minority groups [8,24]. However, instead of adjusting risk values, it cautions clinicians that results may underestimate or overestimate risk depending on the patient’s racial background [25,26]. More recent Canadian guidelines for primary care clinicians, published by the PEER (patients, experience, evidence, research) group, reference the use of both the ASCVD and Framingham calculators [25].

This study aimed to retrospectively investigate and compare cardiovascular risk calculated using both FRS and ASCVD calculators in a randomly selected sample of patients from a single family medical center. The objective was to determine whether individuals prescribed statins based on CVD risk would fall into a different risk category requiring an alternative management approach if risk were estimated using another calculator. The study retrospectively reviewed a random sample of patients prescribed statins for dyslipidemia over the past 10 years, based on Canadian cardiovascular risk guidelines published in 2012. The results were presented as an oral presentation at Western University’s Department of Family Medicine Research Day in June 2024.

## Materials and methods

Study design and setting

A retrospective chart review was conducted at a single family medicine center in London, Ontario.

Population

Patients included were adults aged 40 and older who underwent a lipid assessment between 2010 and 2023. Patients were excluded if they had a statin-indicated condition or prior cardiovascular event. Additionally, patients under age 40 were not included, as not all guidelines and risk calculators were validated in this demographic.

Data extraction

**Figure 1:**
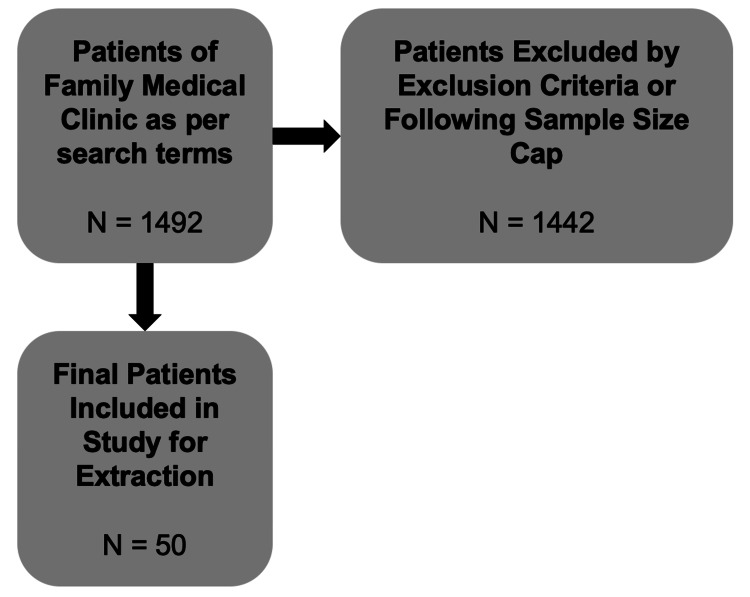
Data abstraction process In total, 1,492 patients were identified from the search, and 50 randomly selected patients were included.

Eligible patients were identified in the electronic medical record software according to search terms including age, statin medication names, serum lipid panel testing, and the year testing was completed. A total of 1,492 records were retrieved from this data query. Following this, charts were manually reviewed and excluded as per the exclusion criteria (Figure 1). The patient’s age at the time of lipid assessment, biological sex, ethnicity (listed as White, Black, or other), blood pressure, total cholesterol, low-density lipoprotein, high-density lipoprotein, date of testing, smoking status (current, former, or never), and presence of treated hypertension were extracted. If, at the time of the cholesterol blood testing, a risk estimation was calculated, this value was also recorded. The data were then re-entered into risk calculation tools deemed readily available online and frequently accessed by clinicians. These calculators included the ASCVD+ calculator [26,27] and the PEER lipid cardiovascular decision aid [28].

Sample size

A sample size of 50 patients was determined to be sufficient. A comparison of values produced by two different risk estimators was planned with a paired t-test. However, the sample size calculation was conducted according to an unpaired t-test to accommodate a larger sample size and achieve a power of 90%. The effect size was assumed to be 5%, which would represent a large enough difference between the two risk scores to potentially shift between risk levels as per the Canadian Dyslipidemia Guidelines of low (<10%), intermediate (10-20%), and high (>20%) risk for a major cardiac event in 10 years. With a value of N=12.59, the noncentrality parameter was equal to √N×E/SΔ, or 3.55, degrees of freedom were 11.5857, and beta was equivalent to 0.0999. This indicated that the sample size in one group of an unpaired t-test would be 13. Doubling this for a paired t-test yielded a sample size of 26. Accounting for an increase in the study sample’s standard deviation (which was not known), this estimated size was doubled again, bringing the final total to approximately 50. There was no missing data in obtaining the patient sample.

Results

The following results were reported: patient characteristics based on CVD risk factors; the proportion of patients with an ASCVD risk differing from their Framingham risk; the proportion of patients who would fall into a different risk category (i.e., <10% versus 10-20%) when using the ASCVD calculator; the mean difference with confidence interval in 10-year cardiovascular risk between the Framingham risk calculators from the PEER CVD decision tool and the built-in electronic medical record calculation documented in the patient’s chart; and the mean difference in 10-year cardiovascular risk between the Framingham risk calculation and the ASCVD risk calculation according to the PEER CVD decision tool. No secondary or subgroup analyses were performed.

Statistical testing

A paired t-test was utilized to determine whether there was a difference in risk between the Framingham risk values documented in the patient’s chart and those calculated using the PEER CVD decision tool. A p-value of <0.05 was considered statistically significant. A paired t-test was chosen because the analysis aimed to identify any overall difference rather than a specifically higher or lower estimation of risk.

## Results

In total, 50 patients were included in this study. Demographic characteristics of the patients are presented in Table 1. A total of 40 of 50 patients (80%) had a CVD risk value (Framingham risk score) documented in their electronic medical record. Overall, 44 of 50 (88%) patients were initiated on statin therapy, while the remaining six were flagged for reassessment.

**Table 1:** Demographic characteristics HDL, high-density lipoprotein

Characteristics	Sub-characeteristics	Number/mean (percentage of total)
Biological sex		
	Male	27 (54%)
	Female	23 (46%)
Age		57.7±1.2 years
Ethnicity		
	White	46 (92%)
	Black	0 (0%)
	Other	4 (8%)
Smoking		
	Current	10 (20%)
	Former	6 (12%)
	Never	34 (68%)
Treated hypertension		16 (32%)
Systolic blood pressure		134±2 mmHg
Aspirin therapy		2 (4%)
Total cholesterol		6.1±0.1 mmol/L
HDL cholesterol		1.3±0.1 mmol/L
Started on Statin		44 (88%)

**Figure 2:**
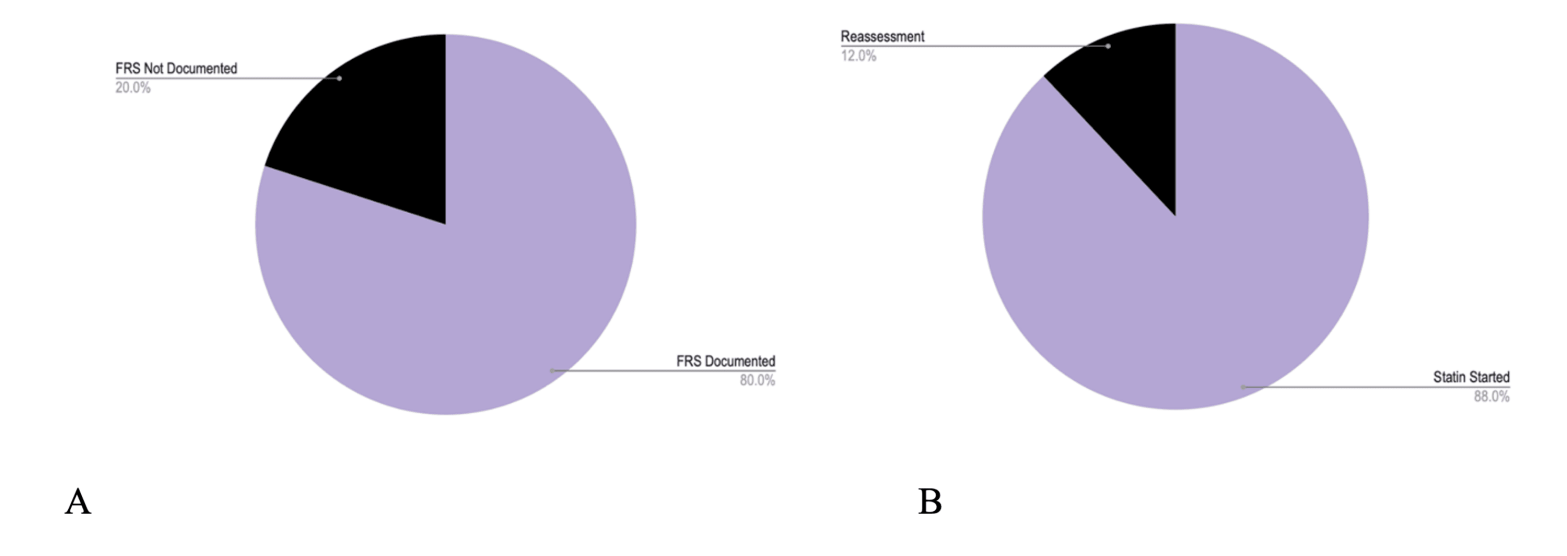
FRS documentation and statin initiation A. Forty of 50 patients had an FRS documented in the chart. B. Forty-four of 50 patients were initiated on statin therapy. FRS, Framingham risk score

The proportion of patients with an ASCVD risk score that differed from their Framingham risk score was 50 of 50 (100%) (Figure 2A). The proportion of patients with an ASCVD risk score that was lower than their Framingham risk score was 49 of 50 (98%) (Figure 2B).

**Figure 3:**
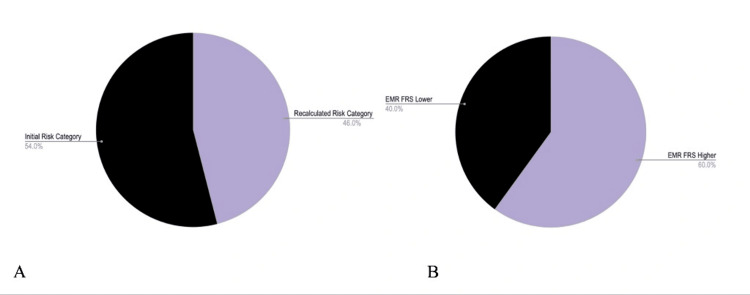
Depending on the risk calculator used, patients would be placed in alternative risk categories according to Canadian guidelines A. Twenty-three of 50 patients would have fallen into a different risk category had the ASCVD score been used instead of the FRS. B. Twenty-four of 40 (60%) patients with documented FRS had EMR FRS values higher than recalculated FRS (via the PEER tool).

According to the Canadian clinical practice guidelines, the proportion of patients who would have fallen into a different risk category if the ASCVD score had been used instead of the Framingham score was 23 of 50 (46%) (Figure 3A). Furthermore, in 24 of 40 (60%) patients, Framingham risk scores reported in the electronic medical record were higher than the recalculated scores using the PEER tool (Figure 3B).

The mean difference in Framingham risk values between the electronic medical record and the PEER lipid online calculator was statistically significant (3.44%, 95% CI 0.11-6.76, p<0.05). The mean difference between Framingham and ASCVD risk values recalculated using the PEER lipid online calculator was also statistically significant (6.83%, 95% CI 5.45-8.21, p<0.0001).

## Discussion

Understanding the results of this study can generate hypotheses and inform future work. It can be inferred that the Framingham risk calculators in the electronic medical record may not accurately reflect a patient’s true cardiovascular risk when compared with other risk scores. Furthermore, depending on the website or calculator used at the point of care, clinicians may obtain significantly different risk values that they, in turn, report to their patients. Given this variance, clinical practice guidelines should clearly state a preference and rationale for which specific tools (and websites) should be used. A comparable example is that of Osteoporosis Canada. In 2023, updated guidelines clearly stated the use of the FRAX risk assessment tool for bone health, with a specific website and standardized, universally used calculator to facilitate this [29]. At present, there are two dyslipidemia guidelines available in Canada, one from the Canadian Cardiovascular Society and another from the PEER group in Alberta [8,25]. With the emergence of the PEER lipid group decision aid tool, this may be a calculator that future national guidelines consider recommending, given its ease of use and quick accessibility during a clinical encounter.

Careful consideration was given to the sample size. Initially, to detect an important difference of 5%, used as a reference for potentially placing a patient in a different risk category, the sample size would have been small (fewer than 20 patients to detect a difference). Accounting for error and debate in selecting this difference, the sample size was essentially quadrupled to include 50 patients in total. The sample was also randomized to ensure blinded patient selection.

The most important finding from this research is that depending on which risk calculator a clinician uses, whether online (across different websites) or through an electronic medical record tool, the results obtained may differ and affect clinical management decisions. For primary care providers, this may be less relevant for patients at intermediate versus high risk for CVD, as patients in both categories may be offered statin therapy. However, if a patient is at low risk with one calculation and moderate risk with another, they may instead be offered a trial of lifestyle management alone rather than pharmacotherapy. This would mean that they are managed differently and may not need to take a medication that otherwise would have been prescribed.

It is important to note that certain calculators have been modified to account for “hard versus soft” outcomes, in other words, outcomes that matter more to patients. Hard outcomes include more objective measures, such as serum cholesterol levels, whereas soft outcomes include events such as having a heart attack. This may explain the variation among calculators used. The assumption was that despite subtle differences in hard and soft outcomes, risk calculators considered the same overall adverse events when assessing a patient’s CVD risk. However, it remains unclear whether a patient’s cardiovascular risk estimated by the ASCVD calculator can be fully extrapolated to the risk categories established by the Canadian Cardiovascular Society guidelines, which rely specifically on the Framingham risk score. Future research could further examine the rationale behind why these risk scores categorize patients differently.

This study has limitations. There is likely some selection bias, as the patient demographics included predominantly white patients in the intermediate cardiovascular risk category. Regarding race, the ASCVD calculator does not alter the percentage risk when selecting “other” race, but does for patients who are African American [24,26]. It notes that estimates may be under- or overestimated depending on race [24,26]. Because of these demographic limitations, the results are less applicable to Canada’s diverse population and more relevant to the demographics of the local family medicine center. Future studies may examine risk estimation and statin prescribing prospectively rather than retrospectively, thereby strengthening validity and generalizability.

Given that 80% of randomly selected patients had a documented risk score in their chart, the standard of care would suggest that 100% of patients should have these calculations completed during physician interpretation to make guideline-informed treatment decisions. However, it is unclear whether this documentation rate is higher or lower than that of other family medicine centers in Canada. Future quality improvement initiatives may aim to increase this percentage to ensure that all patients who have a lipid panel ordered and fall within this demographic receive appropriate follow-up analysis. Finally, ongoing research is focusing on developing clinical decision aids that incorporate outcomes meaningful to patients or are easier to understand, such as their cardiovascular age [30].

## Conclusions

Cardiovascular risk percentages differ between those calculated using an electronic medical record tool and those calculated using online calculators. Cardiovascular risk also varies depending on the specific calculator used, such as the Framingham Risk Score or ASCVD calculator. Depending on the tool, a proportion of patients may fall into a different cardiovascular risk category, leading to different management decisions. Further research, including prospectively designed studies, is required to ensure consistency among point-of-care risk tools. With the emergence of novel risk calculators, updates to clinical practice guidelines should reflect their appropriate clinical applications. Statin prescribing should involve shared decision-making, incorporating patient preference after a comprehensive assessment of individual risk factors.
